# Folic acid inhibits nasopharyngeal cancer cell proliferation and invasion via activation of FRα/ERK1/2/TSLC1 pathway

**DOI:** 10.1042/BSR20170772

**Published:** 2017-12-05

**Authors:** Zhibiao Liu, Xin Jin, Wen Pi, Shouhou Liu

**Affiliations:** Department of Otorhinolaryngology – Head and Neck Surgery, Huai’an First People’s Hospital, Nanjing Medical University, Huai’an, Jiangsu 223300, P.R. China

**Keywords:** Folic acid, folate receptor alpha, invasion, nasopharyngeal cancer, proliferation, TSLC1

## Abstract

Folic acid (FA), which is necessary for normal cell division of mammals, has been implicated to be involved in many tumors. Dietary FA intake has been reported to be associated with a lower risk of nasopharyngeal cancer (NPC). However, the molecular mechanisms of FA in NPC cells remain unclear. In the present study, we found that FA treatment dose dependently inhibited the proliferation, invasion and migration of NPC cells, via folate receptor α (FRα). We further found that FA, bound to FRα, induced the activation of MEK/ERK1/2, and increased the expressions of TSLC1 and E-cadherin. Moreover, blocking of ERK1/2 activation attenuated FA-mediated increase in TSLC1 expression. In addition, knockdown of TSLC1 abolished the FA-mediated inhibition of cell proliferation, invasion and migration, and suppressed the FA-mediated increase oinE-cadherin expression in NPC cells. Taken together, our data suggest that FA treatment inhibits NPC cell proliferation and invasion via activation of FRα/ERK1/2/ TSLC1 signaling pathway. Therefore, FA could be explored as a therapeutic drug for the treatment of NPC, and TSLC1 may act as a tumor suppressor in NPC.

## Introduction

Nasopharyngeal cancer (NPC) is the most common cancer arising from the epithelium of nasopharynx, and has a high incidence in Southern China [1]. Most NPC cases are undifferentiated, and have lymph node metastases [2,3]. NPC is characterized by its highly invasive and metastatic tendencies, and the prognosis is very poor once metastasis occurs [4]. Thus, it is important and urgent to uncover the molecular mechanisms involved in the invasion and metastasis of NPC, and to find new drugs that can improve the prognosis of NPC patients.

Folate is a water-soluble B vitamin that can not be synthesized by mammals and must be obtained from dietary or supplementary sources. Lee et al. [5] have found that lower serum folate is associated with the development and invasiveness of gastric cancer, but Wang et al. [6] have shown that increased serum folate levels have potentially harmful effects on the risk of prostate cancer, suggesting that folate may exert diverse roles in different cancers. Folic acid (FA), a fully oxidized monoglutamyl form of folate, is frequently used as nutritional supplement for folate, since it is more stable and its bioavailability as compared with folate [7]. It is reported that dietary FA intake is protective for NPC in a high-risk population of Chinese adults [8]. Folate receptor α (FRα), a single chain glycosyl-phosphatidylinositol-anchored membrane protein, is the major transporter of FA and acts as an important mediator in many cancers [9]. Studies have reported that FA inhibits endothelial cell proliferation through activating the FRα [10], and inhibits colon cancer cell proliferation through activating the FRα/c-SRC pathway [11]. However, the molecular mechanisms of FA and FRα in NPC progression remain elusive.

In the present study, we investigated the role of FA in NPC progression. Its possible downstream targets on cell proliferation, migration and invasion of NPC cells were also assessed. We finally identified that FA, bound to FRα, inhibited NPC cell proliferation, invasion and migration via ERK1/2 pathway mediated increase in TSLC1 expression.

## Materials and methods

### Reagents

FA and U0126 were purchased from Sigma–Aldrich (St. Louis, MO). Antibodies specific for E-cadherin, β-actin, ERK1/2, MEK, p-MEK, TSLC1 and FRα were obtained from Santa Cruz Biotechnology, Inc. (Santa Cruz, CA, U.S.A.). An antibody specific for p-ERK1/2 (rabbit polyclonal antibody) was obtained from Cell Signaling Technology, Inc. (Beverly, MA, U.S.A.).

### Tissue samples

Tissue samples of NPC were obtained from 20 patients, who had undergone biopsy at the Huai’an First People’s Hospital and were pathologically diagnosed as NPC between January 2010 and December 2015. The study was approved by Nanjing Medical University. All tumor tissues we used were under the approval of the Ethics Committees of Nanjing Medical University.

### Cell culture

Human NPC cell line HONE1 was purchased from Chinese Academy of Medical Science (Beijing, China). The cells were grown in RPMI 1640 culture medium supplemented with 10% FBS in a humidified incubator (37°C, 5% CO_2_).

### Cell count assay

Cells were seeded into a 24-well culture plate at a concentration of 1.0 × 10^4^ cells/well in triplicate, and the cells were treated with or without FA for 144 h. Finally, cells were trypsinized and suspended in cell culture medium, and were counted with a hemocytometer.

### CCK-8 assay

Cells at the density of 0.6 × 10^3^ cells/well were seeded into a 96-well culture plate. Cells were treated with or without FA for 144 h. Then 10 μl of CCK-8 was added into the plate and incubated for 2 h. The plate was shaken for 15 min in dark and the color intensity was measured at 490 nm absorbance using a microplate reader (Bio–Rad Laboratories).

### Invasion assay

Cell invasion assay was performed with a 24-well transwell chamber (Corning Costar, New York, U.S.A.). In brief, the chamber was covered with 30 μl matrigel (BD Biosciences, U.S.A.) to create an artificial basement membrane before the invasion assay was carried out. Cells were pretreated with FA for 1 h. Then, the upper transwell chamber was added with cells (5 × 10^5^ cells) that were suspended in 200 μl serum-free 1640 medium, whereas the lower transwell chamber was filled with 600 μl RPMI-1640 medium supplemented with 20% FBS. Cells were allowed to incubate for 20 h at 37°C, and then the non-invading cells were removed with a sterile cotton swab. The invaded cells were stained with 0.1% Crystal Violet for 20 min at room temperature. The number of invaded cells were counted under a light microscope in seven random fields.

### Migration assay

Cell migration assay was performed with a 24-well transwell chamber (Corning Costar, New York, U.S.A.). In brief, after treating with FA for 1 h, the upper transwell chamber was added with cells (5 × 10^5^ cells) that were suspended in 200 μl serum-free 1640 medium, whereas the lower transwell chamber was filled with 600 μl RPMI 1640 medium supplemented with 20% FBS. Ten hours later, the non-migrating cells were removed with a sterile cotton swab. The migrated cells were stained with 0.1% Crystal Violet for 20 min at room temperature. The number of migrated cells were counted under a light microscope in seven random fields.

### Real-time PCR

Total RNA from cells was extracted using TRIzol reagent (Invitrogen, CA, U.S.A.). Then cDNA was obtained by reverse-transcription PCR with RNA and M-MLV reverse transcriptase (Promega, CA, U.S.A.). Real-time PCR was performed with ABI Prism 7700 sequence detection system (Applied Biosystems, Foster City, CA). The PCR reaction was set as a denaturation step at 95°C for 10 min, then 40 cycles of 95°C for 15 s, and 60°C for 1 min. Primer sequences of E-cadherin, TSLC1, and β-actin were used as follows: E-cadherin sense: 5′- ATTTTTCCCTC GACACCCGAT -3′; E-cadherin antisense: 5′- TCCCAGGCGTAGACCAAGA -3′. TSLC1 sense: 5′- GTCCCACCACGTAATCTGATG-3′; TSLC1 antisense: 5′- CCACCTCCGATTTGCCTTTTA-3′. β-actin sense: 5′-AGAAGGATTCCTATGTG GGCG-3′; β-actin antisense: 5′-CATGTCGTCCCAGTTGGTGAC-3′. All mRNA expressions were normalized to internal control *β-actin* mRNA and relative fold changes were determined by 2^–ΔΔ*C*^_t_ method.

### Western blot

Cells were harvested with RIPA buffer with protease inhibitor and phosphatase inhibitor. Protein concentration was determined by BCA assay (Bio–Rad Laboratories, Hercules, CA). Twenty micrograms of protein was resolved by SDS/PAGE, and then transferred on to PVDF membrane. Then the membrane was blocked with 5% BSA for 1 h at room temperature, and hybridized with antibodies of FRα (1:1000), E-cadherin (1:500), TSLC1 (1:1000), β-actin (1:1000), ERK1/2 (1:1000), MEK (1:500), p-MEK (1:500), or p-ERK1/2 (1:1000) at 4°C overnight. The membrane was incubated with horseradish peroxidase conjugated secondary antibody (Zhong Shan Biotech Co. Ltd, Beijing, China) for 1 h at room temperature. The blots were developed by ECL Plus detection system (Applygen Technologies Inc, Beijing, China), and protein bands were analyzed with the software Quantity One (Bio–Rad, Hercules, CA, U.S.A.).

### Transfection of siRNA

For transient transfection of siRNA in HONE1 cells, FRα siRNA (siFRα), siRNA TSLC1 (siTSLC1) and control siRNA (siCon) were purchased from GenePharma (Shanghai, China), and were transfected with Lipofectamine 2000 (Invitrogen, Carlsbad, U.S.A.) when the cell confluence reached ~40%. The knockdown efficiency was determined by Western blot.

### Statistical analysis

Each experiment was performed at least three times. SPSS (SPSS Inc, Chicago, IL, U.S.A.) 17.0 software was used to perform statistical analysis. Values represent the mean ± S.D. Comparisons were subjected to Student’s *t* test or one-way ANOVA. *P*<0.05 was considered to be statistically significant.

## Results

### FA inhibits NPC cell proliferation and invasion

**Figure 1:**
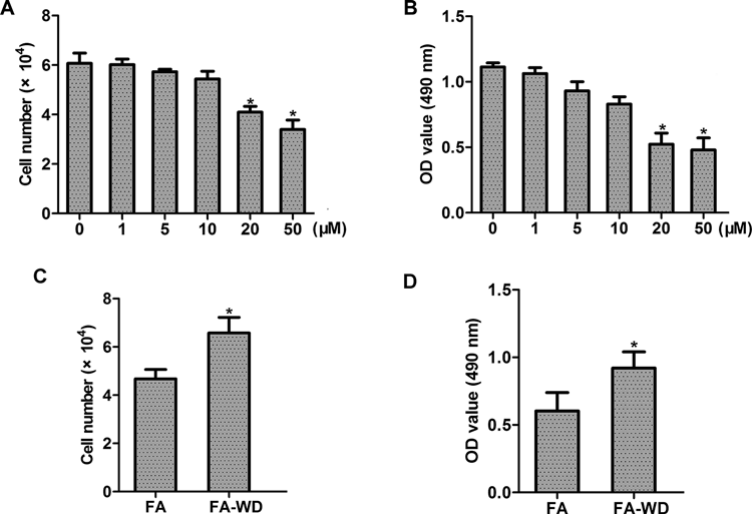
Effect of FA on the proliferation of NPC cells (**A, B**) Effect of FA at a range of concentrations (0–50 μM) on cell proliferation was detected by (A) cell count assay and (B) CCK-8 assay, respectively. (**C, D**) Effect of FA withdrawal on cell growth; *P<0.05. Abbreviation: FA-WD, FA withdrawal.

**Figure 2:**
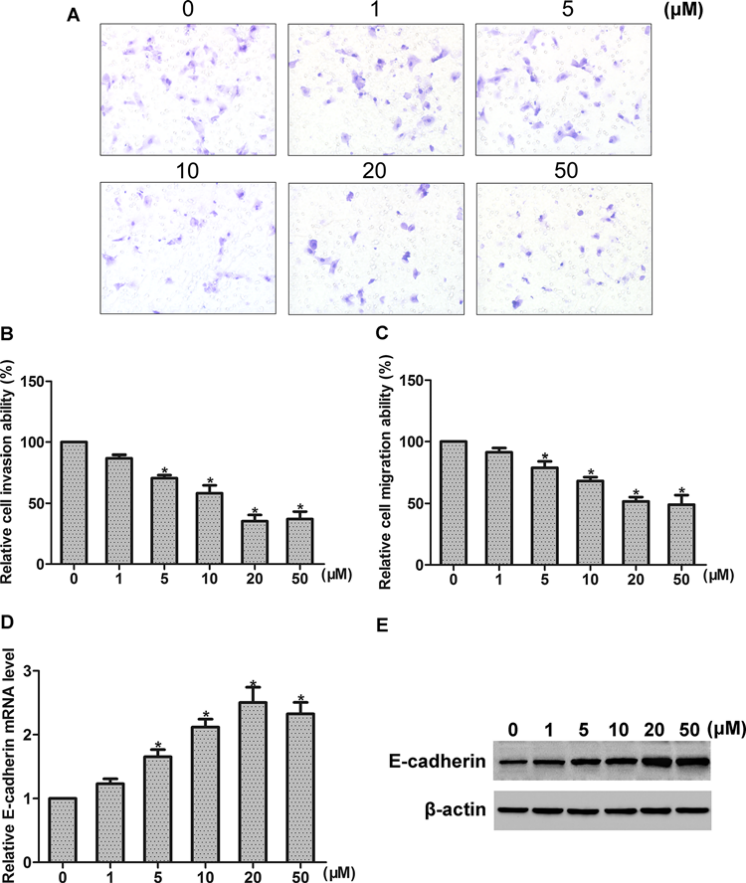
Effects of FA on the invasion, migration, and E-cadherin expression of NPC cells (**A**) Representative photographs of invaded cells that were observed under microscope at ×200 magnification. (**B, C**) Effects of FA at a range of concentrations (0–50 μM) on cell invasion and migration were detected by cell invasion assay and migration assay, respectively. (**D, E**) Effect of FA at a range of concentrations (0–50 μM) on *E-cadherin* mRNA and protein expression was detected by real-time PCR and Western blot analysis, respectively; *P<0.05.

To detect the role of FA in NPC cells, HONE1 cell line was used and different concentrations (0–50 μM) of FA were examined. By cell count assay and CCK-8 assay, we found that FA concentration dependently inhibited the proliferation of HONE1 cells (Figure 1A,B). To further explore whether the effect of FA is reversible, cells were incubated with 50 μM FA for 144 h. Then, FA was withdrawn and cells were incubated with fresh medium for another 36 h. We found that the effect of FA on growth inhibition was reversed without FA incubation (Figure 1C,D). We further examined the effect of FA treatment on NPC cell invasion and migration. By invasion assay and migration assay, we found that FA suppressed the invasion and migration of HONE1 cell, in a concentration-dependent manner (Figure 2A–C). E-cadherin is well known as a key mediator of cancer invasion and metastasis. By real-time PCR and Western blot analysis, we found that FA treatment concentration dependently increased E-cadherin expression of HONE1 cells, at both mRNA and protein levels (Figure 2D,E).

### FA increases activation of MEK/ERK1/2 and expression of TSLC1 in NPC cells

**Figure 3:**
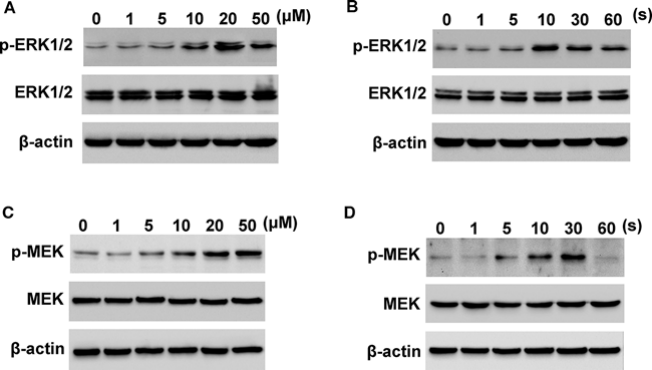
Effects of FA on MEK and ERK1/2 activation (**A**) FA treatment (0–50 μM) dose dependently induced the activation of ERK1/2. (**B**) FA treatment (20 μM) time dependently induced the activation of ERK1/2. (**C**) FA treatment (0–50 μM) dose dependently induced the activation of MEK. (**D**) FA treatment (20 μM) time dependently induced the activation of MEK.

**Figure 4:**
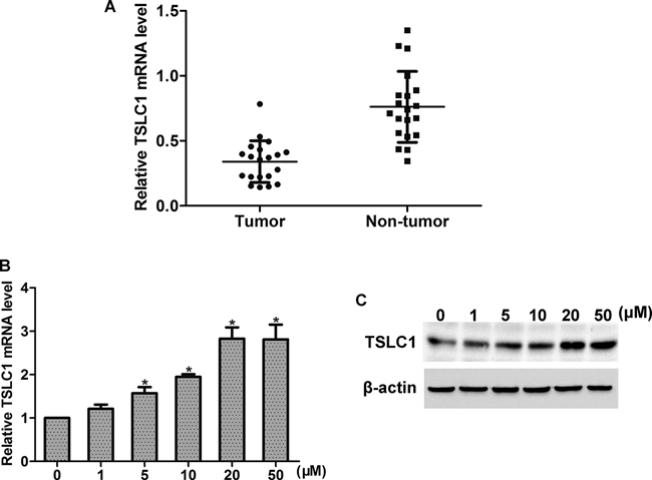
Effects of FA on ERK1/2 activation (**A**) *TSLC1* mRNA level was up-regulated in NPC tissues compared with the corresponding non-tumor tissues. (**B, C**) FA treatment (0–50 μM) dose dependently increased the expression of TSLC1 at mRNA and protein levels; *P<0.05.

MEK/ERK1/2 pathway plays a pivotal role in the progression of cancer. Thus, we here examined the effect of FA treatment on MEK/ERK1/2 activation. Western blot analysis showed that FA dose dependently induced the activation of ERK1/2 in HONE1 cells, with peak activation observed at 20 μM (Figure 3A). Moreover, HONE1 cells were stimulated with FA (20 μM), and ERK1/2 activation was observed in 60 min. The results showed that FA time dependently increased the activation of ERK1/2, with peak activation observed at 10 min (Figure 3B). Moreover, FA stimulated the activation of p-MEK in HONE1 cells, in a dose- and time-dependent manner (Figure 3C,D). Studies have reported that TSLC1 acts as a tumor suppressor in many tumors including NPC [12]. By real-time PCR analysis, we found that the mRNA level of TSLC1 was increased in tumor tissues as compared with that in the corresponding non-tumor tissues (Figure 4A). We then examined the effect of FA on TSLC1 expression. By real-time PCR and Western blot analysis, we found that FA treatment increased the mRNA and protein levels of TSLC1 in HONE1 cells, in a dose-dependent manner (Figure 4B,C).

### Knockdown of FRα alters FA-mediated cell proliferation and invasion

**Figure 5:**
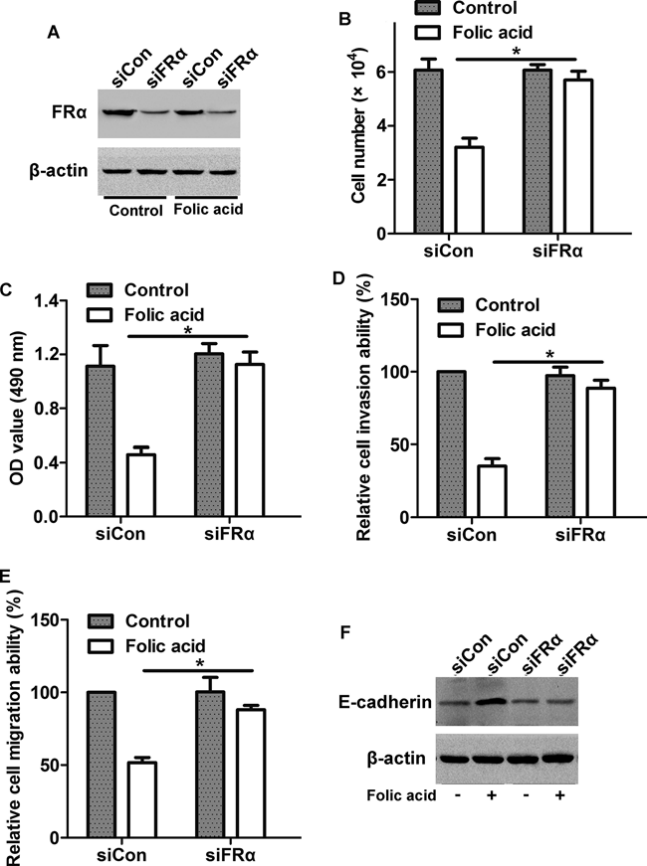
Involvement of FRα in FA-induced decrease in cell proliferation and invasion (**A**) Knockdown efficiency of FRα in HONE1 cells. Cells were transfected with siFRα or siCon for 36 h, and then treated with or without FA for 20 h, the protein level of FRα was detected by Western blot. (**B**) Cell count assay and (**C**) CCK-8 assay showed that transfection with siFRα inhibited the FA-induced decrease in cell proliferation. (**D**) Cell invasion assay and (**E**) migration assay showed that transfection with siFRα inhibited the FA-induced decrease in cell invasion and migration. (**F**) Western blot analysis showed that transfection with siFRα inhibited the FA-induced increase in E-cadherin expression; *P<0.05.

To determine the role of FRα in FA-mediated cell invasion and proliferation, cells were transfected with siFRα to silence the expression of FRα in HONE1 cells. Western blot analysis showed that FRα expression was greatly suppressed in siFRα cells as compared with siCon cells, no matter with or without FA stimulation (Figure 5 A). CCK-8 proliferation assay showed that after knockdown of FRα, the FA-induced decrease in cell proliferation was blocked (Figure 5B,C). Moreover, knockdown of FRα prevented the FA-induced decrease in cell invasion and migration (Figure 5D,E) and increase in E-cadherin expression (Figure 5F). These results suggest that FA blocks NPC cell proliferation, invasion, and migration via activating FRα.

### Knockdown of FRα alters FA-mediated ERK1/2 activation and TSLC1 expression

**Figure 6:**
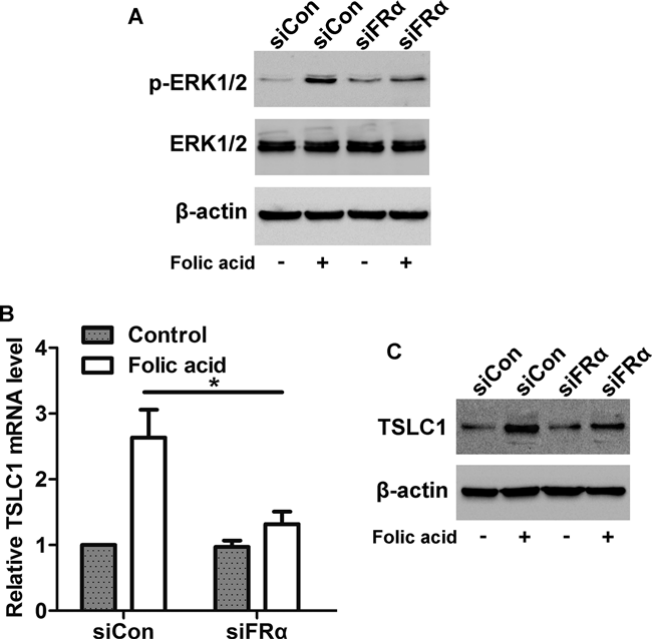
Effects of FRα on FA-induced activation of ERK1/2 and up-regulation of TSLC1 (**A**) Western blot analysis showed that transfection with siFRα inhibited the FA-induced activation of ERK1/2. (**B**) Real-time PCR and (**C**) Western blot analysis showed that transfection with siFRα inhibited the FA-induced up-regulation of TSLC1 at mRNA and protein levels; *P<0.05.

Further, we found that cells transfected with siFRα inhibited FA-induced activation of ERK1/2 (Figure 6A), indicating that FA stimulates ERK1/2 pathway via FRα. We also found that knockdown of FRα blocked the FA-induced increase in *TSLC1* mRNA and protein expression (Figure 6B,C), suggesting that FRα is required for FA-mediated increase in TSLC1 expression.

### Involvement of ERK1/2 pathway in FA-mediated increase in TSLC1 expression

**Figure 7:**
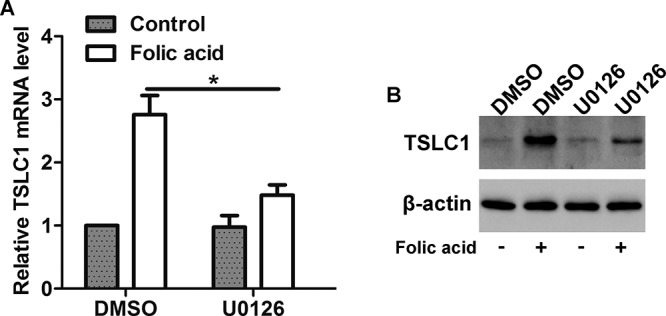
Effect of ERK1/2 pathway on FA-induced up-regulation of TSLC1 (**A**) Real-time PCR and (**B**) Western blot analysis showed that inhibition of the HONE1 cells with U0126 suppressed the FA-induced up-regulation of TSLC1 at mRNA and protein levels; *P<0.05.

To investigate whether the FA-induced increase in TSLC1 expression was regulated by ERK1/2 pathway, HONE1 cells were pretreated with U0126 (an ERK1/2-specific inhibitor, 100 nM) before FA stimulation. As illustrated in Figure 7A, FA stimulation increased the mRNA level of TSLC1 in DMSO-treated cells, whereas the FA-mediated TSLC1 expression was suppressed in U0126-treated cells. Similarly, pretreatment of cells with U0126 inhibited the FA-induced increase in TSLC1 protein expression in HONE1 cells (Figure 7B). These data suggest that ERK1/2 pathway is involved in the FA-induced increase in TSLC1 expression.

### Knockdown of TSLC1 attenuates the effects of FA on NPC cell proliferation and invasion

**Figure 8:**
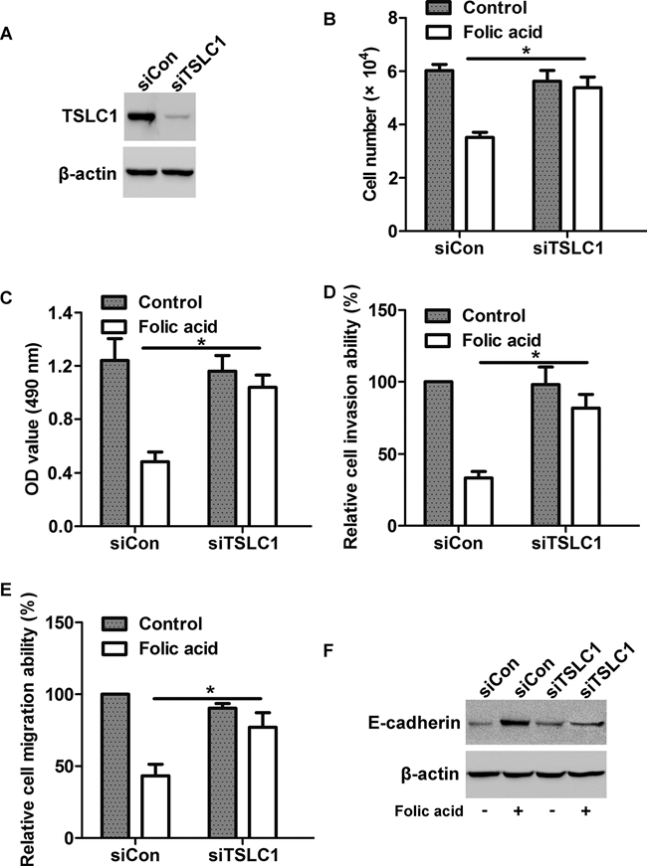
Involvement of TSLC1 expression in FA-induced decrease in cell proliferation and invasion (**A**) Cells were transfected with siTSLC1 or siCon for 48 h, the protein level of TSLC1 was detected by Western blot. (**B**) Cell count assay and (**C**) CCK-8 assay showed that transfection with siTSLC1 attenuated the FA-induced decrease in cell proliferation. (**D**) Cell invasion assay and (**E**) migration assay showed that transfection with siTSLC1 attenuated the FA-induced decrease in cell invasion and migration. (**F**) Western blot analysis showed that transfection with siTSLC1 suppressed the FA-induced increase in E-cadherin expression; *P<0.05.

To determine the effect of TSLC1 on FA-induced decrease in cell proliferation and invasion, cells were transfected with siTSLC1 to silence TSLC1 expression before FA treatment. Western blot analysis showed that transfection with siTSLC1 greatly suppressed TSLC1 expression in HONE1 cells (Figure 8A). By cell count assay and CCK-8 assay, we found that FA treatment inhibited cell proliferation in siCon cells, whereas silencing of TSLC1 prevented the effect of FA on HONE1 cells (Figure 8B,C). By invasion assay and migration assay, we found that knockdown of TSLC1 suppressed FA-induced decrease in cell invasion and migration (Figure 8D,E). Moreover, Western blot analysis showed that the FA-induced increase in E-cadherin protein level was abolished after knockdown of TSLC1 expression (Figure 8F). Taken together, these results implicate that TSLC1 is the key molecule involved in FA-induced proliferation and invasion of NPC cells.

## Discussion

FA has been reported to show diverse roles in different cancers. It is reported that FA inhibits EGFR-mediated proliferation in human colon cancer cells [13], whereas FA treatment increases *in vitro* growth and invasiveness of prostate cancer cells [14]. In the present study, we uncover a negative role of FA in NPC cell proliferation and invasion.

Many studies have reported that FA treatment can suppress the growth of colon cancer cells [15]. In our study, we found that FA dose dependently inhibited the proliferation of NPC HONE1 cells. Wang et al. [16] have reported that FA deprivation enhances invasiveness of human colon cancer cells via epithelial–mesenchymal transition (EMT) process. However, Bistulfi et al. [17] have found that dietary folate deficiency blocks prostate cancer progression in the mouse prostate model. The functions of FA in the progression of NPC are not clear. Here, we found that FA treatment dose dependently inhibited the invasion and migration of NPC HONE1 cells. FA acts its functions via binding to FRα. It is reported that FA can inhibit COLO-205 colon cancer cell proliferation through activating the FRα/c-SRC/ERK1/2/NFκB/TP53 pathway [11]. In our study, using RNAi technology, we found that FA exerted its inhibition effects on NPC cell invasion and proliferation via activating FRα.

MAPK ERK1/2 pathway plays an important role in tumor progression. ERK1/2 has a pleiotropic effect on tumor progression. The intensity of ERK signaling, negative feedback loops, and cross-talks with other signaling pathways, seem to determine the final tumor cellular outcome (promotion or suppression of tumor progression). Studies have found that FA stimulates ERK1/2 phosphorylation in fetal neural stem cells [18], and inhibits COLO-205 colon cancer cell proliferation through activating ERK1/2 pathway [11]. In this study, we found that FA treatment stimulated the activation of ERK1/2 in NPC cells, in a dose- and time-dependent manner. Furthermore, we found that knockdown of FRα suppressed FA-mediated activation of ERK1/2, implying that FA stimulated ERK1/2 activation of NPC cells via FRα.

E-cadherin is a well-known mediator that has a pivotal role in cell–cell adhesion and epithelial development. E-cadherin expression is found to be greatly decreased in many tumors including NPC cells [19]. Pellis et al. [20] have shown that high FA increases E-cadherin expression in human HT29 colon cancer cells, and Wang et al. [16] have found that FA deprivation decreases the expression of E-cadherin. Here, we found that FA treatment dose dependently up-regulated the expression of E-cadherin. After knockdown of FRα, the FA-mediated increase in E-cadherin was inhibited, suggesting that FA increases E-cadherin expression of NPC cells via activating FRa.

TSLC1 has been reported as a key tumor suppressor in many tumors. Down-regulation of TSLC1 expression correlates with poor prognosis in patients with colon, bladder, and ovarian cancer [21–23]. Studies also have shown that TSLC1 is implicated in the regulation of proliferation, invasion, cell cycle, apoptosis, and tumorigenicity in cutaneous and laryngeal squamous cell carcinoma [24,25]. In NPC, it is reported that TSLC1 is significantly associated with lymph node metastases [12]. In our study, we found that TSLC1 was down-regulated in NPC tumor tissues as compared with the corresponding non-tumor tissues. FA treatment dose dependently increased the expression of TSLC1 in NPC cells via FRα/ERK1/2 pathway. Moreover, knockdown of TSLC1 attenuated FA-mediated inhibition of cell invasion and proliferation, and suppressed FA-mediated increase in E-cadherin expression.

In conclusion, our study demonstrates that FA treatment inhibits the proliferation, invasion, and migration of NPC cells, possibly via activating FRα/ERK1/2/TSLC1 pathway. Thus, FA may be crucial for the treatment of NPC.

## Abbreviations

FA: folic acid
FRα: folate receptor α
NPC: nasopharyngeal cancer
siCon: control siRNA
siFRα: FRα siRNA
siTSLC1: TSLC1 siRNA
